# Highly Sensitive Detection and Discrimination of Cell Suspension Based on a Metamaterials-Based Biosensor Chip

**DOI:** 10.3390/bios16010050

**Published:** 2026-01-08

**Authors:** Kanglong Chen, Xiaofang Zhao, Jie Sun, Qian Wang, Qinggang Ge, Liang Hu, Jun Yang

**Affiliations:** 1 Hangzhou International Innovation Institute, Beihang University, Hangzhou 311115, China; 2 Department of Neurosurgery, Peking University Third Hospital, Beijing 100191, China; 3 Center for Precision Neurosurgery and Oncology, Peking University Health Science Center, Beijing 100191, China; 4 Beijing Advanced Innovation Center for Biomedical Engineering, School of Biological Science and Medical Engineering, Beihang University, Beijing 100191, China

**Keywords:** terahertz metamaterials, microcavity, biosensor, cell suspension, living cell, cancer detection

## Abstract

Metamaterials (MMs)-based terahertz (THz) biosensors hold promise for clinical diagnosis, featuring label-free operation, simple, rapid detection, low cost, and multi-cell-type discrimination. However, liquid around cells causes severe interference to sensitive detection. Most existing MMs-based cell biosensors detect dead cells without culture medium (losing original morphology), hindering stable, sensitive multi-cell discrimination. Here, a terahertz biosensor composed of a microcavity and MMs can be used to detect and discriminate multiple cell types within suspension. Its detection mechanism relies on cellular size (radius)/density in suspension, which induces effective permittivity (*ε*_eff_) differences. By designing MMs’ split rings with luxuriant gaps, the biosensor achieves a theoretical sensitivity of ~328 GHz/RIU, enabling sensitive responses to suspended cells. It shows a robust, increasing frequency shift (610–660 GHz) over 72 h of cell apoptosis. Moreover, it discriminates nerve cells, glioblastoma (GBM) cells, and their 1:1 mixture with obviously distinct frequency responses (~650, ~630, ~620 GHz), which suggests effective and reliable multi-cell-type recognition. Overall, this study and its measurement method should pave the way for metamaterial-based terahertz biosensors for living cell detection and discrimination, and this technology may inspire further innovations in tumor investigation and treatment.

## 1. Introduction

Gliomas are the most common and deadliest type of primary intracranial tumor [1]. Glioblastoma (GBM) is the most common and dangerous adult glioma. It is classified as grade IV by the World Health Organization (WHO), with the highest risk of death. It is reported that only about 5% of patients with GBM can survive for about five years [2]. Common first-line treatments include chemotherapy, radiotherapy, and surgical resection, during which image-guided strategies are often applied to distinguish normal and cancerous tissues. However, the margin between normal and cancerous tissue is not clear even under the most advanced imaging systems, which makes it very difficult to effectively and completely remove the cancerous tissue. Tissue biopsy is the gold standard for identifying cancerous tissue. However, it usually takes several days, which is not suitable for instant surgery or radiography therapy. Other quick techniques for the discrimination of multi-cell types, including flow cytometry and magnetic absorption, require fluorescent or immunolabelling techniques, which are expensive, time-consuming, and laborious [3,4]. The fast and effective approach for the quick recognition of cancerous cells, especially neurons, is still in pursuit. Fortunately, biosensors based on MMs working in the terahertz (THz) regime meet this requirement.

Metamaterial-based THz biosensors have caught researchers’ attention due to their great potential in biological research [5,6,7,8,9,10,11,12]. In recent years, many MMs-based THz biosensors have been reported. A novel biosensor based on electromagnetic-induced transparency-like (EIT-like) metamaterial has been employed to detect different densities and apoptosis of oral cancer cells [6]. An EIT metamaterial biosensor has been used for the detection and quick molecular typing of glioma cells [7]. A biosensor based on plasmonic toroidal meta-surface has been proposed for rapid detection and distinction of lung cancer cells [9]. An optically controlled terahertz ultrafast meta-surface has been used as a sensor to achieve high precision for monitoring the cancerous process of gastric cells [10]. In these reports, it takes time for cells to seed on the surface of biosensors, and cells often die during the test. Thus, the measurement results may lose biological significance and cannot realize fast, in-time detection. Another way to detect cells is to deposit cell suspension on the surface of MMs, dry it in a nitrogen environment, and test [12]. Similarly, cells also die during the test, but this method can test cells quickly and in time.

Here, we propose a microcavity and MMs combined biosensor to detect cells in a living state. The MMs consist of a cross and four split rings with luxuriant gaps. The microcavity forms a liquid pool, and the cell suspension is shifted into the pool; then it is integrated with MMs to form a biosensor chip. The MMs can generate a dipole resonance at 1.71 THz, and the theoretical sensitivity of the biosensor is 328 GHz/RIU (RIU, refractive index unit). The effective permittivity of the cell suspension has been theoretically analyzed by Maxwell–Garnett theory, and theoretical inference supports our study. The biosensor has been fabricated and employed to detect GBM cell apoptosis and discriminate between different types of cells. In cell apoptosis detection, the measurement results of the biosensor agree with the commercial method and follow the theoretical inference. More critically, the biosensor has successfully discriminated different types of cells—nerve cells, GBM cells, and mixed cells. Also, the theoretical inference supports the measurement results. Successfully detecting GBM cell apoptosis can help investigate tumor cell apoptosis and distinguish different types of cells, which is beneficial to finding tumor margins and diagnosis, demonstrating significant promise in tumor investigation and treatment via this biosensor, which is low-cost, simple, and fast.

## 2. Design and Theoretical Foundation

### 2.1. Biosensor Chip Design

**Figure 1:**
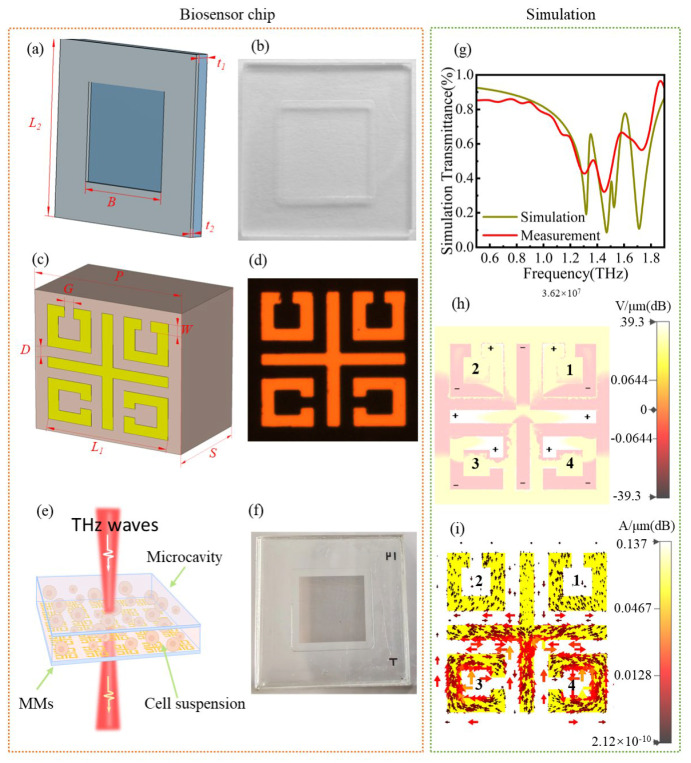
Components of the biosensor chip. The sketch map (**a**) and picture (**c**) of the microcavity. The sketch map (**b**) and fabrication (**d**) of the unit cell in MMs. The sketch map of terahertz waves passing through the bio-chip (**e**) and the picture of the bio-chip (**f**) The image of the integrated microcavity and metamaterials integration. (**g**) Simulation and measurement of transmittance curves. Electric field distribution (**h**) and surface current distribution (**i**) at 1.71 THz.

The biosensor consists of a microcavity and MMs, as shown in Figure 1. The liquid analyte is shifted into the microcavity by pipette (Eppendorf, Hamburg, Germany) and then assembled with MMs. The atmospheric pressure can make two polished quartz plates bind with each other, which can avoid liquid leaking [13,14,15,16]. Figure 1a,b show the sketch map and picture of the microcavity, respectively. The parameters are as follows: *t*_1_ = 1 mm, *B* = 10 mm, *L*_2_ = 18 mm. The depth of the pool is *t*_2_ = 300 μm, which ensures that THz waves can propagate through the liquid, even if the analyte is a polar liquid like water [17,18,19].

The unit cell of MMs is shown in Figure 1c. The substrate is quartz with a thickness of *S* = 1000 μm; the metal is gold with a thickness of 200 nm, and the parameters are as follows: *P* = 80 μm, *W* = 6 μm, *D* = 9 μm, *L* = 66 μm, *D* = 6 μm, *G* = 5 μm. The split rings and cross-combined structure are named SRCCS, and Figure 1d shows the scanning electron microscope of a single unit cell. Figure 1e,f show the schematic diagram and picture of the biosensor chip. This structure was reported in a previous work; the resonance at 1.47 THz was selected as the characteristic peak [12]. This study selects the resonance at 1.71 THz as the characteristic peak. Figure 1g shows the transmittance curves. The envelope of the measurements follows the simulation very well, except that a resonance peak at ~1.51 THz with a high Q-factor was not detected. The substrate thickness will limit the scanning time to detect this sharp resonance peak [20,21]. Fortunately, this resonance peak will not impact our biosensor experiment. Figure 1h shows that the normalized electric field will reveal the physical mechanism. The split rings are named ring1, ring2, ring3, and ring4 in an anticlockwise direction. The normalized electric field indicates that there are six pairs of dipoles. The weak dipoles are on ring1 and ring2, the stronger two are on ring3 and ring4, and the strongest is on the cross. The electric field direction of the incident THz wave is in the *y*-direction, and the structure is symmetric according to the *y*-axis. Coincidentally, these dipole distributions are symmetric according to the *y*-axis at 1.71 THz. Though the electric field distribution seems complicated, on the whole, it excites a dipole resonance [22,23,24]. The surface current distribution will further confirm that. The corresponding surface current in Figure 1i indicates that the dipoles in split rings are magnetic dipoles, thanks to the current in each near split ring being in the opposite direction. The neighboring magnetic dipole is always in the opposite direction [20]. The surface current along the upper *y*-axis and right *x*-axis (and along the lower *y*-axis and left *x*-axis) also excites two magnetic dipoles [9,25,26]. Hence, the magnetic dipole excites the resonance at 1.71 THz.

The theoretical sensitivity of biosensors based on this structure has been numerically calculated. The calculation method is described in our previous work [12]. The numerical calculation of the minimum thickness of the analyte layer to ensure the MMs perform best as a sensor is about 15 μm, and the theoretical sensitivity is 328 GHz/RIU. That means when the thickness of the analyte ≥ 15 μm, the biosensor can achieve the best performance.

### 2.2. Theoretical Foundation for Cell Detection in Suspension

The cell suspension contains cells and the host medium—phosphate-buffered saline (PBS). The radius of Na^+^, K^+^, HPO_4_^2−^, and H_2_PO_4_^−^ in PBS solution is on the hundred-nanometer scale, which is far away compared to the cell radius [27]. Thus, only cells and water are counted in the effective dielectric constant *ε_eff_* calculation. The *ε_eff_* can be described as follows [28]:

(1) $$\varepsilon_{\mathrm{eff}}\;=\;\varepsilon_{c}V\;+\;\left(1\;-\;V\right)\varepsilon_{w}\;=\;\varepsilon_{w}\;+\;(\varepsilon_{c}\;-\;\varepsilon_{w})V$$

Here, *ε_w_* and *ε_c_* are the dielectric constants of water and the cell, respectively. *V* is the volume fraction, *V* = (4π/3)*Nd*^3^, *N* is the cell number, and *d* is the cell radius.

In the frequency range from 0.5 THz to 3 THz, the permittivity of water and cell satisfies the relationship *ε_c_* < 4.5 < *ε_w_* < 5.5 [29,30,31]. Therefore, *ε_c_* < *ε_w_*. According to Equation (1), we can theoretically infer the following: (a) When the density increases, *ε_eff_* of cell suspension will decrease for the same cell type. (b) For the different types of cells under the same density, the suspension within cells whose diameter is bigger will have a lower *ε_eff_*. The permittivity change will result in vibration of the transmission curve of MMs.

## 3. Method

**Figure 2:**
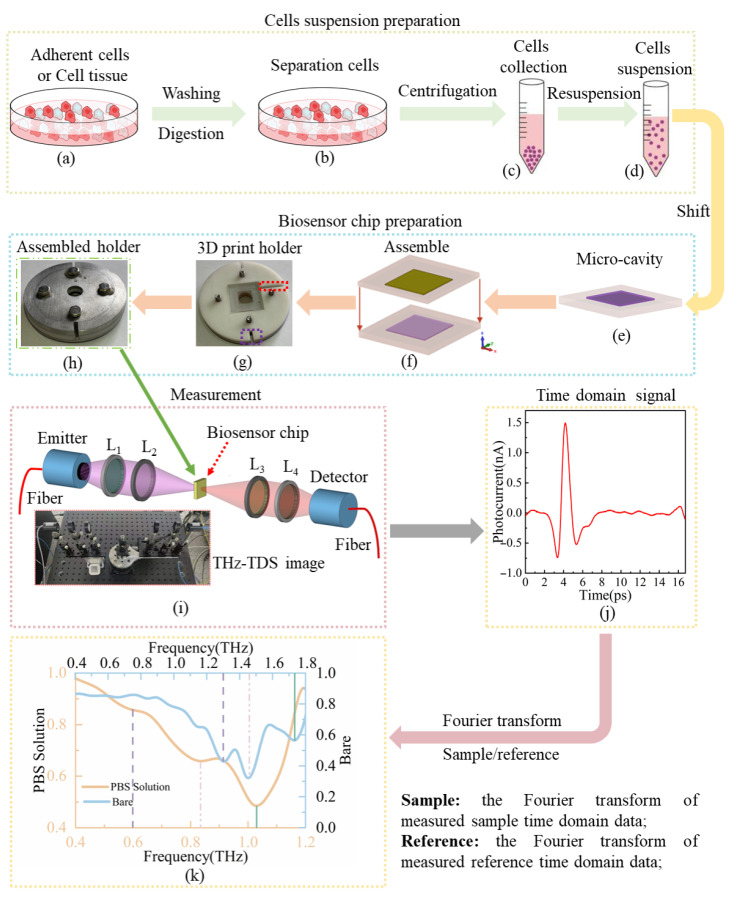
The flow of cell suspension, biosensor preparation, biosensor measurement, and the comparison of measurements for bare MMs and the PBS solution. (**a**) Cells cultured in a culture dish. (**b**) Digested cells (or cell tissue). (**c**) Collected cells. (**d**) Prepared cell suspension. (**e**) Schematic diagram of the microcavity loaded with cell suspension. (**f**) Schematic diagram of microcavity and metamaterials integration. (**g**) Integrated microcavity-metamaterials assembly mounted on the holder (the purple-marked gap served as a directional indicator, while the red-marked channel was designed to drain excess suspension overflowing during the assembly of the microcavity with metamaterials). (**h**) The assembled biosensor chip. (**i**) Schematic diagram and image of the THz-TDS system. (**j**) Time-domain curve of terahertz signals. (**k**) Transmittance of the biosensor chip with and without PBS.

In this study, the cell suspensions were shifted to the microcavity of the biosensor chip. The experimental workflow for cell suspension, biosensor chip preparation, and measurement is shown in Figure 2. The details are described as follows:

### 3.1. GBM Cell Preparation

The GBM cells (Peking Union Medical College) are cultured by the traditional method [32]. Once GBM cells are cultured, they are washed with PBS, as shown in Figure 2a. After that, the adherent cells are digested by 0.25% trypsin–EDTA (HyClone, GE Healthcare, Logan, UT, USA), and the digested cells are centrifuged and collected, as shown in Figure 2b,c. Finally, the collected GBM cells are rewashed with PBS, and then prepared into a GBM cell suspension with a density of 1 × 10^5^ cells/mL, as shown in Figure 2d.

### 3.2. Nerve Cell Preparation

The nerve cells are harvested from ICR mice (Peking University Health Science Center). The ICR mice are killed and dissected, and the brain tissue is taken (Details regarding animal ethics approval are provided in the Supplementary Document). The brain tissue is washed with PBS and digested with 0.25% trypsin–EDTA. After that, the digested nerve cells are centrifuged and collected. Then, the cells are rewashed with PBS. Finally, the nerve cells are prepared into a nerve cell suspension with a density of 1 × 10^5^ cells/mL. The graphical workflow maps of these steps are shown in Figure 2a–d.

### 3.3. Biosensor Chip Preparation

The biosensor chip consisted of MMs, a microcavity, and a holder. Firstly, the microcavity, metamaterials, and holder were rinsed with deionized water and then re-rinsed with 75% alcohol. Subsequently, they were dried in a nitrogen environment. The dry holder (Figure 2g) was placed on the experimental table in a dust-free environment. The purple-marked gap served as a directional indicator, while the red-marked channel was designed to drain excess suspension that overflowed during the assembly of the microcavity with metamaterials. Subsequently, the microcavity was placed into the holder, and the suspension was pipetted into the microcavity. Critically, an excess volume of suspension was used to ensure that the biosensor chip cavity was completely filled when the metamaterials were laminated onto the microcavity. Overflowing suspension was channeled through the drainage pathway, preventing contamination of the biosensor chip surface, which may disturb the measurement results. Finally, the holder cover was assembled (Figure 2h).

The terahertz time-domain spectroscopy (THz-TDS, the details of the THz-TDS system are provided in the Supplementary Document) is employed to measure the biosensor [12,33], as shown in Figure 2i. Finally, the domain waveform is obtained, as shown in Figure 2j. Due to the modulator function of the PBS solution, the resonance peak frequencies of the PBS solution measured by the biosensor chip are 0.6, 0.84, and 1.03 THz, while the corresponding frequencies of bare MMs are 1.3, 1.46, and 1.73 THz, respectively, as shown in Figure 2k. The reason that the resonance amplitude of the PBS solution at 0.6 and 0.84 THz can be almost neglected is that the image part of the PBS solution’s *ε*_eff_ is relatively high, which means that most of the THz wave energy in that frequency range (below 1 THz) is absorbed by the solution. The resonance at these specific frequencies cannot be excited effectively [29,34]. Hence, the resonance at 1.71 THz is selected as the characteristic peak.

## 4. Measurement and Discussion

The biosensor is employed to detect GBM cell apoptosis and distinguish different cell types. Theoretical inference and commercial methods support our measurement results. In this study, all measurements were performed with at least three independent replicates.

### 4.1. Sensitive Detection of GBM Cell Apoptosis

In cancer treatment, one of the key indicators is cell apoptosis [1]. Anti-cancer drugs temozolomide (TMZ) can promote cancer cell apoptosis. The cell’s apoptosis is detected by our biosensor and commercial method.

#### 4.1.1. Cell Counting Kit-8 Method

**Figure 3:**
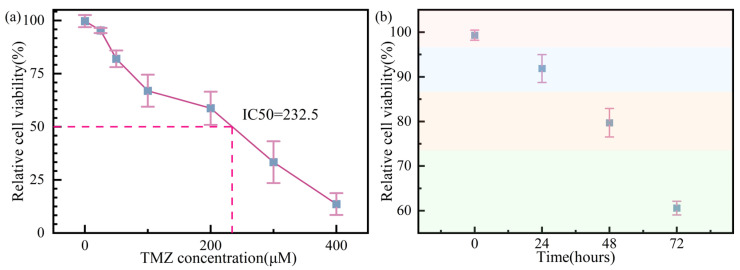
GBM cell apoptosis detection by the CCK-8 method. (**a**) The relative cell viability relationship of TMZ density. (**b**) The relative GBM cell viability under different TMZ action time conditions, detected by the CCK-8 method.

The GBM cell apoptosis is detected by a commercial method. GBM cells were seeded into 96-well plates [35]. Subsequently, different quantities of TMZ are added to the cells to confirm the appropriate concentration of TMZ (Sigma, St. Louis, MO, USA). Figure 3a shows that when the quantity reaches ~200 μM, the relative cell viability is 50%, which means that 200 μM TMZ is the appropriate concentration [36]. After that, TMZ acts on GBM cells at different times, 0, 24, 48, and 72 h, and the results are shown in Figure 3b. The relative cell viability percentages for 0, 24, 48, and 72 h are ~100%, ~91%, ~79%, and ~60%, respectively.

#### 4.1.2. Biosensor Chip Detection Method

Cells have to be labeled and time-consuming in the CCK-8 method, while the biosensor has the advantage of being label-free, fast, and simple. The biosensor chip was employed to detect GBM cell suspensions with different TMZ administration times. The process of GBM cell suspension is shown in Figure 2a–d. Notably, apoptotic cells are significantly more fragile than viable cells; during the aforementioned processing steps (rinsing, digestion, centrifugation, collection, re-rinsing), most apoptotic cells ruptured, with their cell membranes and cytoplasm washed away. Hence, the suspension primarily contained viable cells, with only a small fraction of apoptotic cells remaining. Cell suspensions were not incubated in the microcavity for the entire 72 h duration; rather, suspensions were freshly prepared and measured at each time point. In addition, measurements were performed immediately after suspension preparation to avoid errors caused by temporal variations in the analyte.

**Figure 4:**
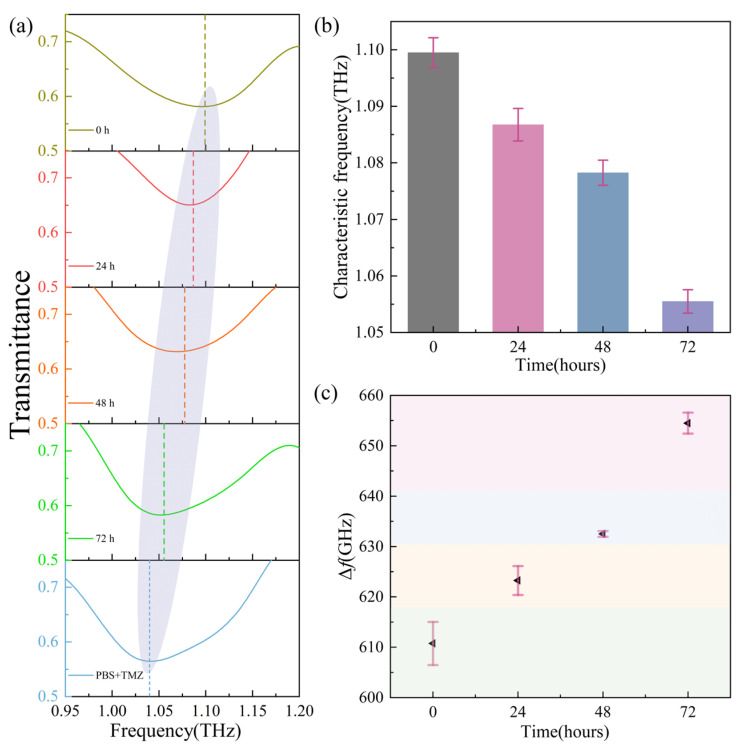
GBM cell apoptosis detection by biosensor chip. (**a**) The transmission curves of PBS + TMZ and different TMZ action times on GBM suspensions. (**b**) The average characteristic peak resonance frequencies with error bar extracted from (**a**). (**c**) The error bar and Δ*f*.

Figure 4a presents the transmission curves of PBS with TMZ and GBM cells under different TMZ action times. The characteristic resonance peak significantly redshifts with the increase in action time. The average value of resonance frequencies extracted from Figure 4a is shown in Figure 4b. The corresponding resonance frequencies of PBS + TMZ, 0, 24, 48, and 72 h are ~1.03, ~1.1, ~1.09, ~1.08, and ~1.06 THz, respectively. The standard deviation method (SD) was employed to quantify measurement errors; the corresponding error bars for the average characteristic frequency of PBS + TMZ at 0, 24, 48, and 72 h are ~±0.26%, ~±0.29%, ~±0.22%, and ~±0.21%, respectively. Figure 4c shows the corresponding Δ*f* values at 0, 24, 48, and 72 h are ~610, ~620, ~630, and ~660 GHz, respectively. The error bars (SD) are ~± 0.43%, ~± 0.29%, ~±0.1%, and ~±0.21%, respectively. From the Δ*f* values and error bars, we can infer that the measurement results are reliable.

Once the suspension was prepared, the cell number in the suspension was quantified using a cell counter (NucleoCounter^®^ NC-200™, ChemoMetec A/S, Allerød, Denmark), with at least three independent replicate measurements. In addition, the density of the GBM cell suspension can be inferred according to Figure 3b. The cell density deduced via the cellular viability method matched the cell counter measurements.

Equation (1) reveals the relationship between density and effective dielectric constant *ε_eff_*. For the same type of cell (each cell size is almost the same), as the density increases, *N* increases, then *V* increases, which will lead to a decrease in *ε_eff_*. The resonance frequency is given by the following:

(2) $$f=\frac{1}{2\pi \sqrt{\mathrm{LC}}}$$

Here, *L* is the inductance, and *C* is the capacitance. In our measurement, changes in *ε_eff_* will lead to changes in the *C* of the biosensor, resulting in transmission spectrum vibration [12]. According to Equations (1) and (2), the characteristic frequency redshifts as the density decreases. The data in Table 1 follow the theoretical inference.

Comparing the measurement results of commercial method and the biosensor, the two results present a relatively good agreement. Moreover, the results also agree with theoretical inference, making the results believable. Our biosensor has successfully detected the GBM cell apoptosis.

### 4.2. Cell Discrimination

To diagnose tumors, it is essential to distinguish between tumor cells and normal cells, including those that are mixed. The biosensor is used to determine different types of cells. The details of the cell suspension are as follows: (a) In the GBM cell suspension (GCS), there are 1 × 10^5^ GBM cells per milliliter. (b) In the nerve cell suspension (NCS), there are 1 × 10^5^ nerve cells per milliliter; (c) In the mixed cell suspension (MCS), there are 5 × 10^4^ nerve cells and 5 × 10^4^ GBM cells per milliliter, and the total cell number is 1 × 10^5^. The density of the three types of cell suspension is 1 × 10^5^ cells/mL.

**Figure 5:**
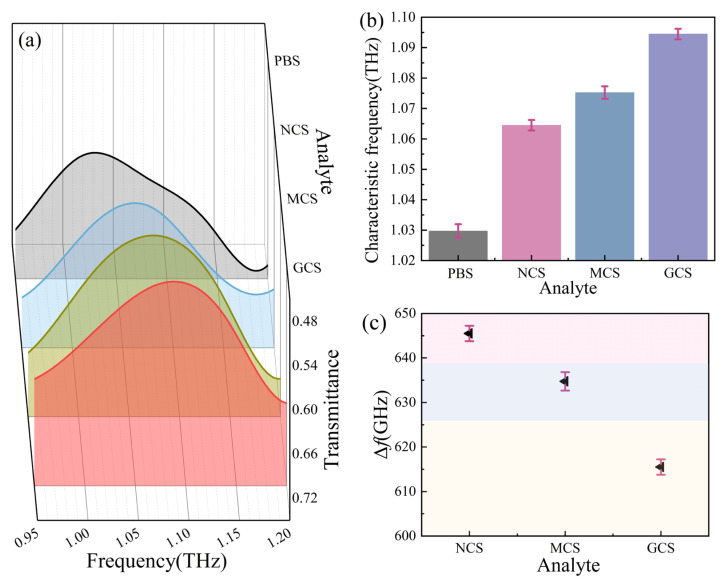
The measurement results of cell discrimination. (**a**) The transmission curves of different types of cell suspensions under a 1 × 10^5^ cells/mL density condition. (**b**) The average characteristic peak resonance frequency with error bars extracted from (**a**). (**c**) The error bar and Δ*f*.

Figure 5a shows the measurement transmission spectra of these cell suspensions in a 3D plot. There is a significant frequency shift between each curve. The characteristic frequencies are extracted and shown in Figure 5b. The average characteristic frequencies of PBS, NCS, MCS, and GCS are ~1.03 ~1.06, ~1.08, and ~1.09 THz, respectively. The corresponding error bars (SD) for PBS, NCS, MCS, and GCS are ~±0.22%, ~±0.17%, ~±0.20%, and ~±0.17%, respectively. The Δ*f* between NCS and GCS is ~30 GHz, which means nerve and GBM cells have been distinguished. Tumor cells at the margin may intermingle with nerve cells; thus, identifying the tumor margin has great significance in tumor resection. Figure 5c shows that the Δ*f* values among NCS, MCS, GCS, and bare MMs are ~650, ~630, and 620 GHz, respectively. The corresponding error bars (SD) are ~±0.17%, ~± 0.21%, and ~±0.17%, respectively. The Δ*f* points with the error bar do not overlap, meaning that the three cell types have been distinguished.

Table 2 presents the cell’s average radius and resonance frequency. The *ε_c_* and cell’s radius affect *ε_eff_*. Based on the inherent biological characteristics of nerve cells and GBM cells, it is well-established that the average radius of nerve cells is smaller than that of GBM cells Based on the inherent biological characteristics of nerve cells and glioblastoma multiforme (GBM) cells, it is well-established that the average radius of nerve cells is smaller than that of GBM cells [37,38,39]. Here, ***d*****1** denotes the average radius of nerve cells, while ***d*****3** denotes that of GBM cells. According to the aforementioned cellular characteristics, the relationship $\bar{d}1$ < ***d*****3** can be inferred. The mixed cell suspension contained equal numbers of nerve cells and GBM cells, thus the average radius of the mixed suspension (***d*****2**) can be approximated by the formula $\bar{d}2$ ≈ (***d*****1** + ***d*****3**)/2. Therefore, direct measurement of the cellular radii was not required, and the quantitative relationship among the three parameters can be deduced as follows: ***d*****1** < ***d*****2** < ***d*****3**, which results in *V*_NC_ < *V*_MC_ < *V*_GC_. The complex permittivity of the cell is given by [40]:

(3) $$\varepsilon_{c}\;=\;\varepsilon_{\mathrm{men}}\Psi$$

(4) $$\Psi =\frac{\gamma^{3}+2(\frac{\varepsilon_{i}-\varepsilon_{\mathrm{mem}}}{\varepsilon_{i}+2\varepsilon_{\mathrm{mem}}})}{\gamma^{3}-(\frac{\varepsilon_{i}-\varepsilon_{\mathrm{mem}}}{\varepsilon_{i}+2\varepsilon_{\mathrm{mem}}})}$$

Here, *γ* = *d*/(*d* − *T_m_*), *d* is the radius of the cell, *T_m_* is the thickness of the cell membrane, and *ε_i_* and *ε_mem_* are the complex permittivity of the cytoplasm and the membrane, respectively. As for *ε_i_*, *ε_mem_*, and *T_m_*, there is little difference between nerve cells and GBM cells. Thus, in Equation (4), *Ψ_NC_* ≈ *Ψ_MG_* ≈ *Ψ_GC_*. Therefore, Equation (3) tells us that *ε_NC_* ≈ *ε_MC_* ≈ *ε_GC_*. The critical factor influencing the *ε_eff_* is volume fraction *V* = (3/4) *πd^3^*. From Equation (1), *ε_eff_* = *ε_w_* + (*ε_c_* − *ε_w_*)*V*, with the *V* increase, *ε_eff_* will decrease due to (*ε_c_* − *ε_w_*) < 0. The decrease of *ε_eff_* will result in characteristic peak resonance frequency increase, which means that for the same density of cell suspension, the effective permittivity will decrease with increasing cell diameter. The data in Table 2 support the theoretical inference. The measurement results indicate that the biosensor has successfully detected different types of cells.

## 5. Application Prospect

The experiments for detecting GBM cell apoptosis and discriminating cell types have been successfully conducted, with encouraging results, including suppression of the proliferation and spreading of tumor cells and promotion of healthy cell proliferation, also helping the immune system to remove defective cells [11,12,13,14]. The biosensor provides a low-cost and simple way to detect cancer cell apoptosis.

**Figure 6:**
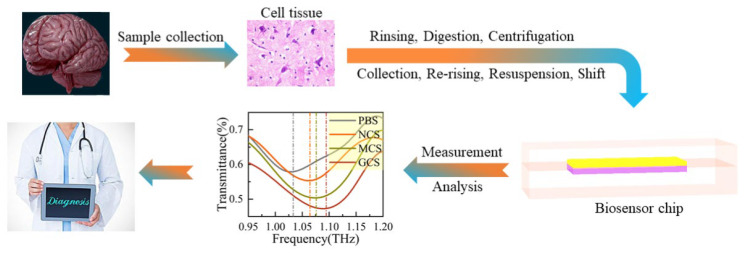
Application prospect.

Chemotherapy, radiotherapy, and surgical resection are the main approaches for cancer treatment [14,15,16]. The first two modalities can induce apoptosis in patients, while the latter involves the surgical removal of tumors from the patient’s body. Tumor cells at the tumor margin are often intermingled with surrounding healthy cells. Therefore, a rapid, accurate, and simple method for detecting mixed cells (a mixture of healthy cells and tumor cells) is in urgent demand for tumor surgical resection. In this study, the proposed biosensor has been proven capable of successfully detecting and distinguishing between healthy and tumor cells. Figure 6 illustrates the process for locating the tumor margin: First, tissue specimens were harvested. Subsequently, the tissue was processed through a stepwise protocol involving rinsing, enzymatic digestion, centrifugation, cell collection, and re-rinsing; finally, a cell suspension with a concentration of approximately 1 × 10^5^ cells/mL was prepared. The suspension is then transferred to the biosensor for testing. This method offers the advantages of label-free detection and operational simplicity. Furthermore, the testing time for the cell suspension is no more than 10 min. Cell discrimination using our biosensor can significantly facilitate the surgical resection of tumors.

## 6. Conclusions

In this study, the apoptosis of GBM cells and discrimination of different types of cells by a THz biosensor based on MMs-microcavity have been reported. The microcavity is designed as a liquid pool to keep cells alive during the test. Through the design of luxuriant gaps in the structure of the biosensor, the sensitivity is significantly improved, which can fulfill the detection and discrimination of multiple cell types within suspension. The theoretical sensitivity of the biosensor is relatively high (328 GHz/RIU) and robust (error variance < 1%) compared with reported studies10,38,39, which means the proposed MMs are promising for cell-based sensing applications. Thus, the cell-based biosensor can be used for long-term cell apoptosis measurement (72 h) while maintaining cell viability, which may be applied in tumor cell apoptosis. Moreover, the NCS, MCS, and GCS cell suspensions have been successfully distinguished using this biosensor with distinct frequency differences (~650, ~630, and ~620 GHz for NCS, MCS, and GCS, respectively), which is promising to be used as a real-time detection in surgical tumor resection to find the margin of the tumor. Overall, this biosensor supplies a new way to realize fast, low-cost, and in-time cell type recognition. The key advantage of this detection is that the cells remain alive, allowing the biological significance to be retained and studied further simultaneously via other techniques.

## Figures and Tables

**Table 1:** The relationship between GBM cells suspension density, TMZ action time, and characteristic resonance frequency.

Action Time (Hours)	0	24	48	72
Density (cells/mL)	~1 × 10^5^	~9 × 10^4^	~8 × 10^4^	~6 × 10^4^
Resonance frequency (THz)	~1.1	~1.09	~1.08	~1.06

**Table 2:** The relationship between cell diameter and characteristic peak frequency.

Analyte	Nerve Cell	Nerve & GBM Cell	GBM Cell
Cell’s radius (μm)	$\bar{d}$1	$\bar{d}$2	$\bar{d}$3
V (μm^3^)	$\bar{V}$ *_NC_*	$\bar{V}$ *_MC_*	$\bar{V}$ *_GC_*
Resonance frequency (THz)	~1.06	~1.08	~1.09

## Data Availability

Data can be accessed upon reasonable request from the corresponding author at klchen@buaa.edu.cn.

## Supplementary Materials

The following supporting information can be downloaded at: https://www.mdpi.com/article/10.3390/bios16010050/s1, Figure S1. The schematic of the image optical modulation angle resolved THz-TDS. References [12,33,41,42,43] are cited in the supplementary materials.

## Abbreviations

The following abbreviations are used in this manuscript:

CCK-8	Cell Counting Kit-8
PBS	Phosphate-buffered saline
Eq.	Equation
GBM	Glioblastoma
GCS	GBM cell suspension
MCS	Mixed cell suspension
MM	Metamaterial
NCS	Nerve cells suspension
RIU	Refractive index unit
SD	Standard deviation
TMZ	Temozolomide
WHO	World Health Organization
