# Supplementary material for: Highly Sensitive Detection and Discrimination of Cell Suspension Based on a Metamaterials-Based Biosensor Chip
Source: Biosensors (Basel). 2026 Jan 8;16(1):50. doi: 10.3390/bios16010050 (PMC12839188; doi:10.3390/bios16010050)
Supplement: Supplementary file 1 [file biosensors-16-00050-s001.zip › biosensors-4014685-supplementary.pdf]

biosensors
MDPI

Supplementary Material

Highly Sensitive Detection and Discrimination of Cell Suspension Based on a Metamaterials-Based Biosensor Chip

Kanglong Chen [1,*,†], Xiaofang Zhao [2,†], Jie Sun [3,†], Qian Wang [4], Qinggang Ge [2], Liang Hu [4,*] and Jun Yang [2,*]

[1] Hangzhou International Innovation Institute, Beihang University, Hangzhou 311115, China
[2] Department of Neurosurgery, Peking University Third Hospital, Beijing 100191, China;
[3] Center for Precision Neurosurgery and Oncology, Peking University Health Science Center, Beijing 100191, China
[4] Beijing Advanced Innovation Center for Biomedical Engineering, School of Biological Science and Medical Engineering, Beihang University, Beijing 100191, China
* Correspondence: klchen@buaa.edu.cn (K.C.); cnhuliang@buaa.edu.cn (L.H.); yangjbysy@bjmu.edu.cn (J.Y.); Tel.: +86-18810650967 (K.C.)
† These authors contributed equally to this work.

1. Animal ethics approval

1.1. Supplementation of Animal Ethics Approval Information

All animal experiments involving ICR mice were strictly reviewed and approved by the **Experimental Animal Ethics Committee of Peking University Third Clinical Medical College**. The official approval number is SA20250288.

All experimental procedures were performed in strict accordance with the **Guide for the Care and Use of Laboratory Animals** and the animal welfare regulations of our institution[1]. The experimental design strictly followed the 3R principles (Replacement, Reduction, Refinement) to minimize the number of experimental animals and reduce their pain and distress during the experiment.

Modification in the manuscript: The above information has been added to the **2.1 Experimental Animals** section of the revised manuscript, and the revised part is marked in red for easy identification.

1.2. Specification of Anesthesia and Euthanasia Methods for Mice

We have supplemented the detailed protocols of anesthesia and euthanasia for ICR mice in the **2.2 Primary Neuron Isolation** section of the manuscript, with the specific description as follows [2]:

For anesthesia, 6~8 weeks-old specific-pathogen-free (SPF) ICR mice were intraperitoneally injected with sodium pentobarbital (50 mg/kg body weight). The depth of anesthesia was monitored by checking the disappearance of the pedal reflex and corneal reflex; only mice with complete loss of reflexes were used for subsequent operations. For euthanasia, after the collection of brain tissues, the mice were subjected to cervical dislocation under deep anesthesia, which is a rapid, painless, and humane method recommended for laboratory rodents, and complies with the euthanasia standards of the American Veterinary Medical Association (AVMA).

Modification in the manuscript: The above content has been added to the corresponding part of the "Materials and Methods" section, and the revised content is marked in red.

We confirm that all animal experimental procedures fully comply with the relevant animal welfare and ethical norms. All revised contents have been integrated into the manuscript, and we have carefully checked the consistency of the entire text. We hope these modifications can well address the reviewer’s concerns.

2. Measurement Setup and Experimental Conditions

The transmission spectra were measured in a self-built THz time-domain spectroscopy (THz-TDS), as shown in Figure S1. The spectroscopy has been mentioned in published papers [3–5].

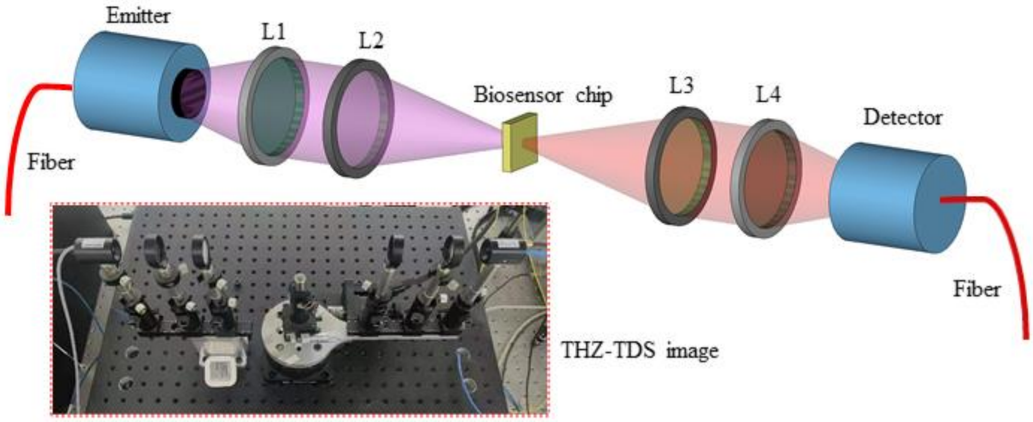

Figure S1. The schematic of the image optical modulation angle resolved THz-TDS.

The TDS system can generate a horizontal polarization THz when a laser drives on the InGaAs photoconductive antenna. The laser is generated by a commercial fiber (FemtoFErb FD 6.5, Toptica Company, Germany), whose pulses are 60 fs, the spectral width is 1550 nm, and the power is 80 mW. There are two types of lenses shown in Fig. 4, collimating lens (L1 and L3) with focal length f=50 mm and condenser lens (L2 and L4) with focal length f=100 mm. Once the THz wave is generated, the wave first comes through L1 and turns to parallel light. Subsequently, the parallel light will focus when it passes through L2, and the sample (biosensor chip) is placed where the focus point is. Then the THz wave, which brings detection messages, passes through L3 and the parallel light propagation to L4. Finally, the THz wave passes through L4, then focuses on the receiver antenna. The receiver antenna transforms THz radiation into a current signal. The THz radiation electric field vector can be detected by scanning optical delay line based on phase-locked amplifier technology. The diameter of the wave spot is about 5 mm, and the periodicity of the unit cell is 80 μm, which means about 5000-unit cells can be radiated by THz radiation. Our experiment's scanning time is 19 ps, and the minimum resolution is 10 GHz. The experiment setup and conditions ensure that our measurement results are reliable and can be repeated.

This study's experiments were carried out in Beijing, China, during January. The THz-TDS system was placed in a sterile, temperature- and humidity-controlled room, and measurements were thus conducted under conditions of ~25 °C and ~30% air humidity.

Reference

1. National Research Council (U.S.). *Guide for the Care and Use of Laboratory Animals*, 8th ed.; National Academies Press: Washington, DC, USA, 2011; ISBN 978-0-309-15400-0.
2. Leary, S.L. (Ed.) *AVMA Guidelines for the Euthanasia of Animals: 2020 Edition*; American Veterinary Medical Association: Schaumburg, IL, USA, 2020; ISBN 978-1-882691-09-8.
3. Chen, K.; Ruan, C.; Zhan, F.; Song, X.; Fahad, A.K.; Zhang, T.; Shi, W. Ultra-Sensitive Terahertz Metamaterials Biosensor Based on Luxuriant Gaps Structure. *iScience* **2023**, *26*, 105781. https://doi.org/10.1016/j.isci.2022.105781.

4. Yang, P.-D.; Ouyang, C.; Hong, T.-S.; Zhang, W.-H.; Miao, J.-G.; Wu, X.-J. Study of phase transition of single crystal and polycrystalline vanadium dioxide nanofilms by using continuous laser pump-terahertz probe technique. *Acta Phys. Sin.* **2020**, *69*, 204205. https://doi.org/10.7498/aps.69.20201188.
5. Xiong, H.; Sun, H.; Zhou, J.; Li, H.; Zhang, H.; Liu, S.; Cai, J.; Feng, L.; Miao, J.; Chen, S.; et al. Terahertz Anisotropy in Fascia and Lean Meat Tissues. *Biomed. Opt. Express* **2022**, *13*, 2605–2615. https://doi.org/10.1364/BOE.454338.
